# Peritoneal Tuberculosis in a Female Patient Mimicking Peritoneal Carcinomatosis: A Case Report

**DOI:** 10.7759/cureus.113359

**Published:** 2026-07-25

**Authors:** Melih E Ceyhan, Sude N Çukurova, Zeynep Tatar, Harun M Guven, Servet Karagul

**Affiliations:** 1 Department of General Surgery, Atlas Üniversitesi, Istanbul, TUR; 2 Department of Pathology, Patomer Private Pathology Laboratory, Istanbul, TUR; 3 Department of Radiology, Atlas Üniversitesi, Istanbul, TUR

**Keywords:** abdominal pain in females, ascite, peritoneal carcinomatosis mimic, peritoneal tuberculosis (ptb), tumor marker ca-125

## Abstract

Peritoneal tuberculosis (PTB) is a rare extrapulmonary disease. The clinical and radiological findings closely resemble those of peritoneal carcinomatosis, leading to diagnostic confusion. Herein, we present a case of PTB in a patient who underwent general surgery with a preliminary diagnosis of peritoneal carcinomatosis. A 47-year-old woman presented with abdominal pain for three months. Abdominal evaluation revealed generalized tenderness and mild defenses. Contrast-enhanced abdominal CT demonstrated free fluid in the peritoneal cavity, especially in the lower abdomen, irregular infiltration of the anterior intraperitoneal fat tissue, and many small nodular lesions on the peritoneal and omental surfaces. The CA-125 level (758.3 U/mL) was markedly elevated. Gynecologic assessment did not reveal any evidence of malignancy. Colonoscopy revealed sigmoid diverticulosis. Upper endoscopy revealed erosive antral gastritis. Due to the persistent diagnostic uncertainty, diagnostic laparoscopy was performed. Biopsies of the omentum and peritoneum were obtained. Histopathological examination revealed widespread granulomatous inflammation with epithelioid histiocytes and Langhans-type giant cells. The QuantiFERON-TB Gold test results were positive. The patient was evaluated in consultation with the Department of Infectious Diseases. Anti-tuberculosis therapy was initiated immediately and has been ongoing without any adverse events for seven months.

## Introduction

Tuberculosis mostly affects the lungs, but it can also affect other parts of the body [1]. Extrapulmonary tuberculosis refers to an infection outside the lungs. It may affect the pleura, lymph nodes, abdominal organs, skin, bones, joints, meninges, and genitourinary system [2]. Peritoneal tuberculosis (PTB) is a rare form of extrapulmonary disease. The clinical and radiological findings closely resemble those of peritoneal carcinomatosis [3]. Diagnosis of this disease is often challenging and usually delayed because the symptoms are non-specific. In addition, radiological, histopathological, and molecular tests may not provide fast or clear results [4]. Clinical symptoms are generally vague and can include abdominal pain, ascites, weight loss, and general systemic complaints [5,6]. Imaging findings such as peritoneal nodules, omental thickening, and abdominal fluid were very similar to those of peritoneal carcinomatosis. PTB can easily be mistaken for advanced ovarian cancer or peritoneal carcinoma because they have very similar clinical and laboratory findings, including ascites, pelvic masses, and high serum CA-125 levels [7]. Herein, we present a case of PTB in a patient who underwent general surgery with a preliminary diagnosis of peritoneal carcinomatosis.

## Case presentation

A 47-year-old woman presented with three months of left-sided abdominal pain and two days of right-sided pain prior to admission. She had a history of ankylosing spondylitis and a previous cesarean section. She also reported a decreased appetite, night sweats, and an unplanned weight loss of approximately 6 kg over the previous two months. On examination, her vital signs were stable: her blood pressure was 125/85 mmHg, her pulse rate was 78 beats per minute, her respiratory rate was 16 breaths per minute, and her temperature was 36.5°C. Abdominal evaluation revealed generalized tenderness and mild defense. Other system examinations were unremarkable. Laboratory tests showed mild normocytic anemia with a hemoglobin value of 11.4 g/dL. The lymphocyte count was low. The C-reactive protein level was increased at 8.1 mg/L. Initial laboratory findings are summarized in Table 1.

**Table 1:** Laboratory findings of the patient

	Result	Reference Range
White blood cell count	4.60 ×10³/µL	4.5–11.0 ×10³/µL
Hemoglobin	11.4 g/dL	11.7–16.0 g/dL
Platelet count	256 ×10³/µL	150–400 ×10³/µL
Absolute neutrophil count	3.39 ×10³/µL	1.8–7.7 ×10³/µL
Absolute lymphocyte count	0.75 ×10³/µL	1.2–3.9 ×10³/µL
C-reactive protein (CRP)	8.1 mg/L	<5 mg/L
Blood urea nitrogen (BUN)	11.5 mg/dL	6–20 mg/dL
Creatinine	0.63 mg/dL	0.5–0.9 mg/dL
Aspartate aminotransferase (AST)	32 U/L	5–34 U/L
Alanine aminotransferase (ALT)	30 U/L	0–55 U/L
Prothrombin time (PT)	10.8 s	10–14 s
International normalized ratio (INR)	0.91	0.8–1.2
Activated partial thromboplastin time (APTT)	21.8 s	22.1–30.2 s
Cancer antigen 125 (CA-125)	758.3 U/mL	<35 U/mL

**Figure 1:**
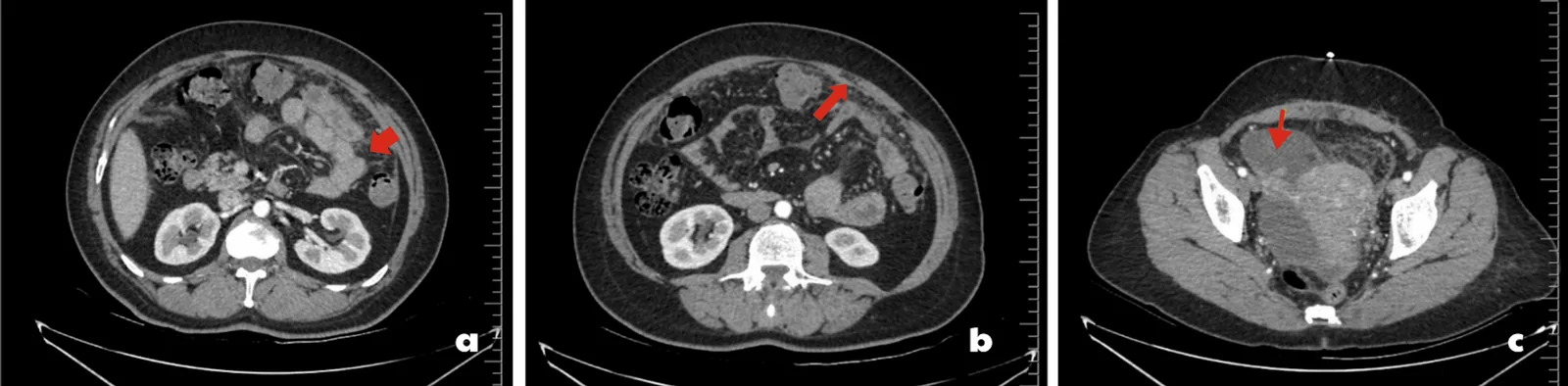
Preoperative CT images of the patient The patient's CT abdominal examination revealed heterogeneity in the omental fatty tissue (a). Peritoneal thickening (b). Free intra-abdominal fluid (c). The findings primarily suggest peritonitis. Differential diagnosis includes peritonitis carcinomatosis.

Abdominal ultrasonography revealed free anechoic fluid in the pelvic area and around the intestinal loops. Contrast-enhanced abdominal computed tomography (CT) demonstrated free fluid in the peritoneal cavity, especially in the lower abdomen, irregular infiltration of the anterior intraperitoneal fat tissue, and many small nodular lesions on the peritoneal and omental surfaces (Figure 1). These findings were considered to be compatible with peritoneal carcinomatosis or peritonitis. Thoracic images showed bilateral pleural effusion, which was more prominent on the left side, and small subpleural lung nodules.

**Figure 2:**
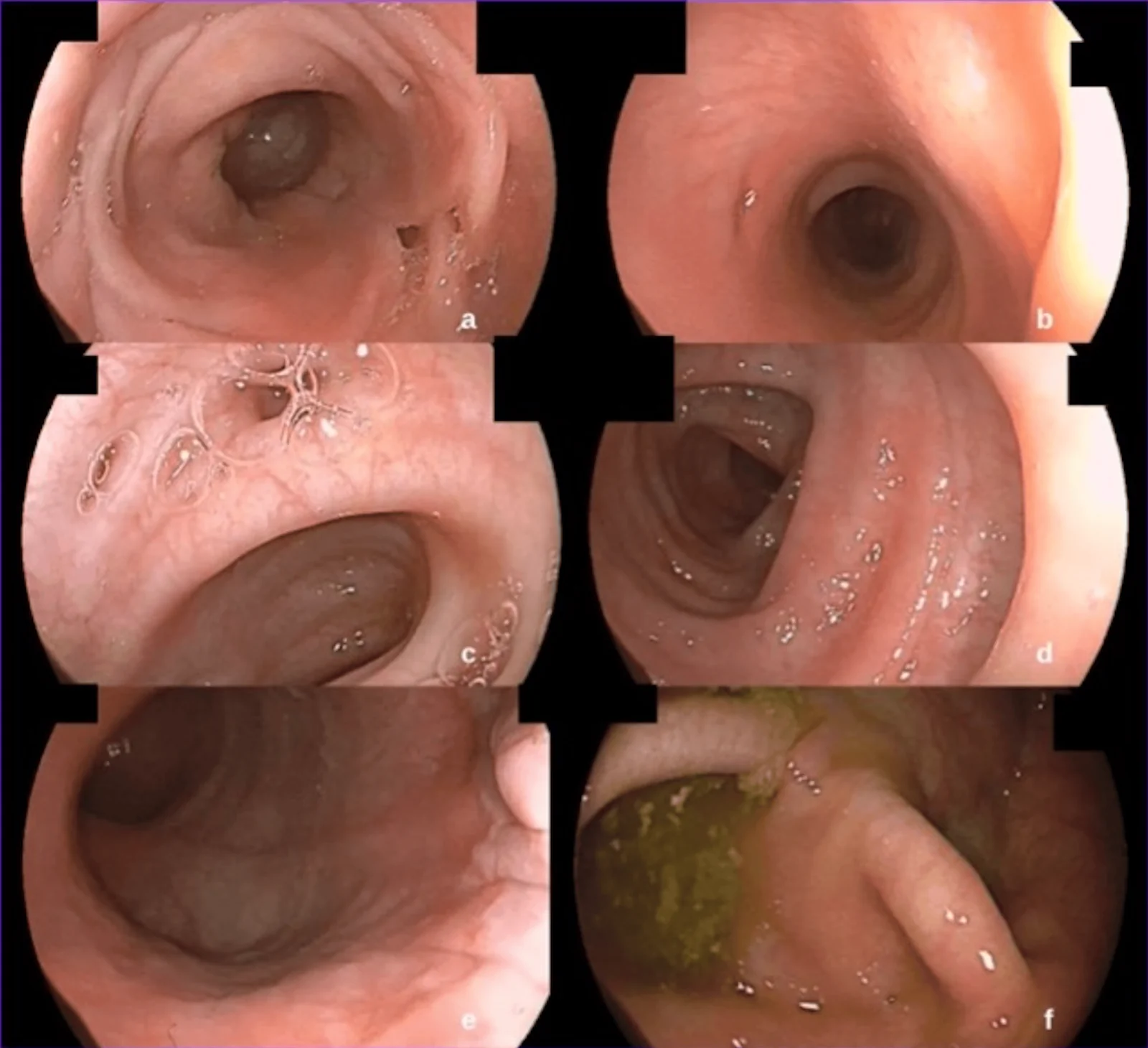
Images of colonoscopy Images of colonoscopy: (a) splenic flexure; (b) ascending colon; (c) sigmoid colon demonstrating diverticular disease; (d) transverse colon; (e) rectum; and (f) cecum. Colonoscopy revealed sigmoid diverticulosis without evidence of malignancy.

**Figure 3:**
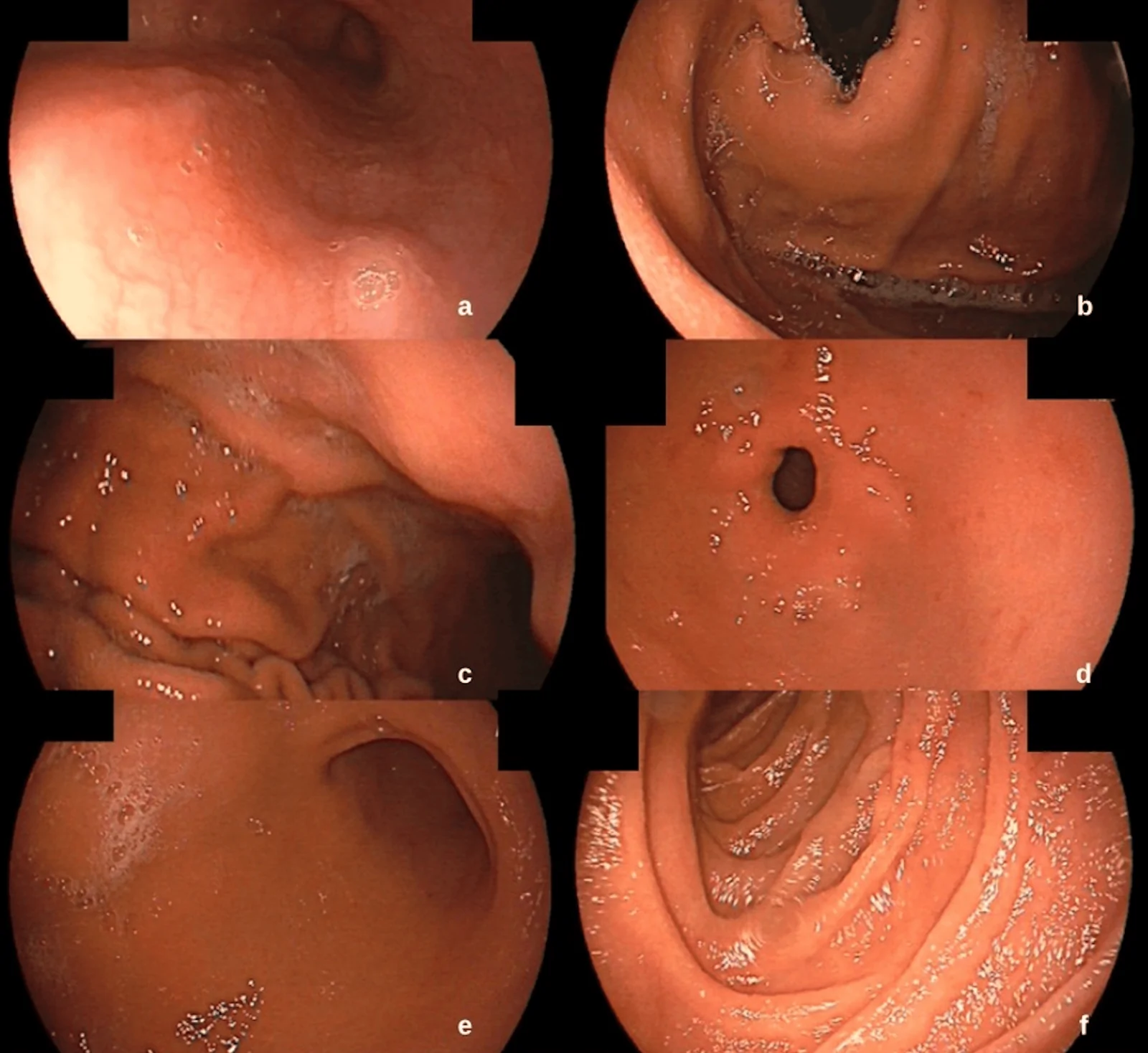
Images of upper endoscopy Images of upper endoscopy: (a) esophagus; (b) cardia; (c) gastric body; (d) pylorus; (e) duodenal bulb; and (f) second portion of the duodenum. Endoscopic examination revealed erosive antral gastritis.

**Figure 4:**
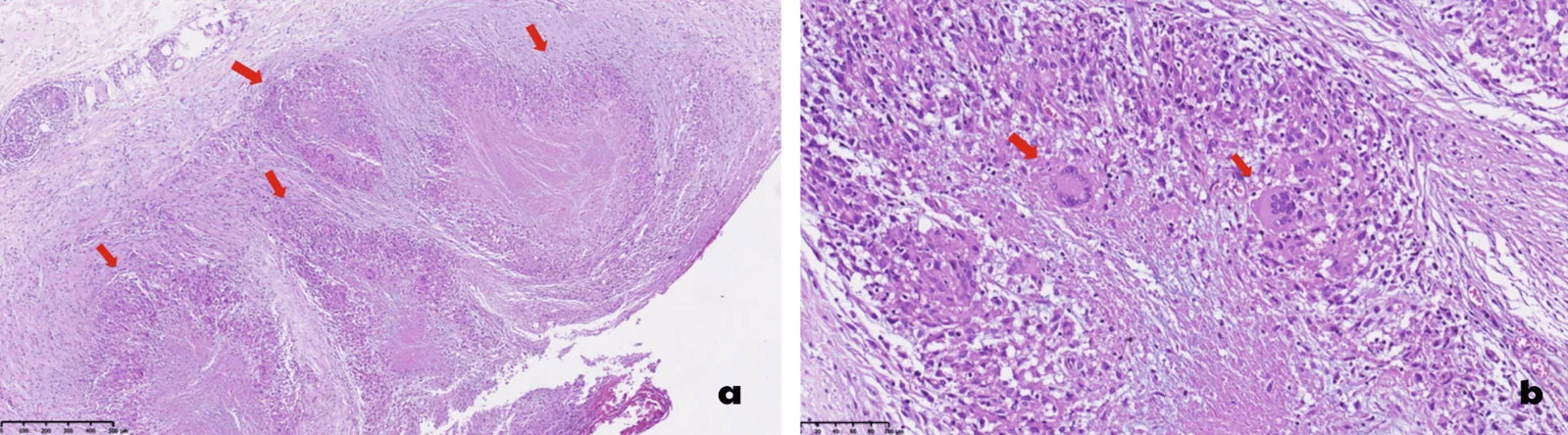
Histopathological examination of the peritoneal biopsy specimen Hematoxylin and eosin (H&E) stained pictures at ×5 magnification show several granulomas of different sizes. The granulomas are separated from each other and marked with arrows. All granulomas have central necrosis (a). A closer view of one granuloma at ×20 magnification. The arrows show two giant cells (Langhans Type). In one cell, nuclei form a horseshoe pattern. In the other cell, the nuclei are grouped on one side of the cytoplasm. Around giant cells, there are epithelioid histiocytes with unclear cytoplasmic borders and large vesicular nuclei (b). Histopathological examination of the peritoneal biopsy specimens shows granulomatous inflammation consistent with tuberculosis. There was no evidence of malignancy.

Considering the imaging results and the markedly elevated CA-125 level (758.3 U/mL), peritoneal carcinomatosis of gynecological origin was thought to be the most probable diagnosis. Gynecologic assessment did not reveal any evidence of malignancy. Colonoscopy revealed sigmoid diverticulosis (Figure 2). Upper endoscopy detected erosive antral gastritis with positive Helicobacter pylori infection (Figure 3). Examination of the ascitic fluid revealed a serous and slightly bloody appearance. No bacterial growth was detected in the culture. Acid-fast bacilli staining and Mycobacterium tuberculosis PCR were negative. Owing to persistent diagnostic uncertainty, diagnostic laparoscopy was performed, and multiple diffuse nodules were observed over the peritoneum and omental surfaces (Figure 4). Biopsies of the omentum and peritoneum were obtained. The patient was discharged on the first postoperative day.

**Figure 5:**
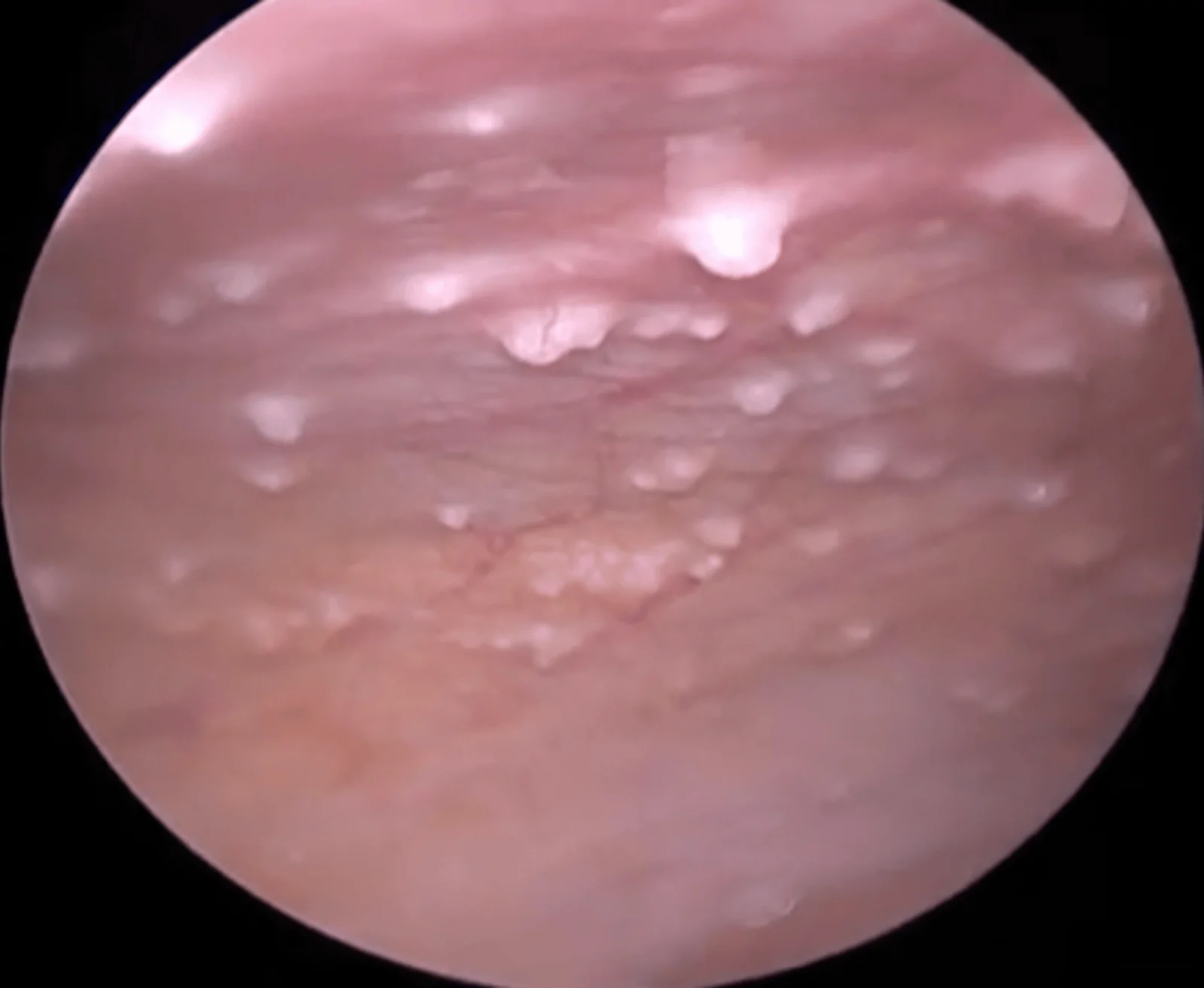
Intraoperative view of the peritoneum Laparoscopic view shows a laparoscopic view demonstrating multiple whitish miliary nodules over the parietal peritoneum, suggestive of peritoneal tuberculosis.

Histopathological examination revealed widespread granulomatous inflammation with epithelioid histiocytes and Langhans-type giant cells. Foci of caseation-like necrosis were also observed. Malignant cells were not observed. Ziehl-Neelsen staining did not reveal definite acid-fast bacilli. These findings were highly suggestive of tuberculous peritonitis (Figure 5). The QuantiFERON-TB Gold test results were positive. The patient received a standard four-drug regimen of isoniazid, rifampicin, pyrazinamide, and ethambutol during the two-month intensive phase, followed by isoniazid and rifampicin for the continuation phase, for a total planned duration of six months of anti-tuberculosis therapy. Weight-based dosing was used per national tuberculosis guidelines. Six months into the treatment, a contrast-enhanced abdominal computed tomography scan revealed no ascites and no abnormal signs. CA-125 levels decreased to 41.2 U/mL. There was an improvement in clinical symptoms. At the seventh postoperative month, the patient was followed up uneventfully and antituberculosis treatment was ongoing.

## Discussion

PTB is difficult to diagnose because it resembles peritoneal carcinomatosis in clinical findings and imaging studies [8]. CA-125 levels are often elevated in cancer, but they can also increase in peritoneal inflammation and tuberculosis [3,8]. CA-125 is a large transmembrane glycoprotein. It is produced by the coelomic epithelium, including the peritoneum, pleura, and pericardium. It is also secreted by the Müllerian epithelium, including the fallopian tubes, endometrium, and endocervix [9]. Negative culture and PCR results in ascitic fluid do not rule out PTB. The bacterial load in a fluid is typically low [10,11]. The tuberculin skin test (TST) and interferon-gamma release assays (IGRAs) may support this diagnosis. However, they cannot differentiate latent infections from active disease [12]. Therefore, they should not be used in isolation. Histopathological confirmation is necessary for definitive diagnosis. Some studies have reported that IGRAs have higher sensitivity and specificity than TST [13]. Radiological imaging commonly shows ascites with peritoneal and omental involvement. Enlarged necrotic lymph nodes are frequently observed in the abdomen. The ileocecal area was the most commonly involved bowel segment. Liver and spleen involvement may present as small abscesses. Adrenal and pancreatic involvement is uncommon [14]. Laparoscopic biopsy is the most reliable diagnostic modality. It allows the direct visualization of peritoneal surfaces and adequate tissue sampling [1,8,15]. This case shows that tuberculosis must always be considered when ascites and peritoneal nodules are detected, particularly in endemic regions.

PTB is a rare but clinically significant type of extrapulmonary tuberculosis. It presents serious diagnostic challenges because it strongly mimics peritoneal carcinomatosis. Ascites, diffuse peritoneal nodules, omental involvement, and markedly elevated CA-125 levels often suggest intra-abdominal cancers. This phenomenon is more common among women. However, these findings are not specific to malignancy. They can also occur under inflammatory conditions of the peritoneum. This case demonstrates that negative microbiological results do not rule out PTB. These tests included acid-fast bacillus staining, culture, and PCR analysis of the ascites fluid. This is particularly important in cases of low bacterial counts. In such cases, relying solely on imaging and laboratory results can lead to delayed or incorrect diagnosis. Histopathological analysis performed via laparoscopic peritoneal biopsy is the most reliable diagnostic tool. It allows direct examination of the peritoneum and provides sufficient tissue for evaluation. In areas where tuberculosis is prevalent or in patients with systemic symptoms such as weight loss and night sweats, PTB should always be considered. It should be included in the differential diagnosis in patients with unexplained ascites and peritoneal thickening. Therefore, an early diagnosis is crucial. It prevents unnecessary oncological treatment and allows early initiation of anti-tuberculosis therapy. This case highlights the importance of maintaining a strong clinical suspicion of PTB in patients with radiological and biochemical findings suggestive of peritoneal malignancy.

## Conclusions

PTB should be considered in the differential diagnosis of patients with peritoneal nodules, ascites, and elevated CA-125 levels, particularly if they live in endemic regions. Radiological and laboratory findings may closely mimic those of peritoneal malignancies. Diagnosis via laparoscopic biopsy enables early initiation of appropriate treatment while preventing unnecessary oncological interventions.
